# Diagnostic contribution of GeneXpert Ultra in pediatric pulmonary tuberculosis

**DOI:** 10.36416/1806-3756/e20240241

**Published:** 2025-03-18

**Authors:** Claudia Stella Pereira Battaglia, Ana Alice Amaral Ibiapina Parente, Ronir Raggio Luiz, Rafaela Baroni Aurilio, Michely Alexandrino de Souza Pinheiro, Maria de Fátima Bazhuni Pombo Sant’Anna, Clemax Couto Sant’Anna

**Affiliations:** 1. Instituto de Puericultura e Pediatria Martagão Gesteira, Universidade Federal do Rio de Janeiro, Rio de Janeiro (RJ), Brasil.; 2. Faculdade de Medicina, Universidade Federal do Rio de Janeiro, Rio de Janeiro (RJ), Brasil.

**Keywords:** Pulmonary tuberculosis, children, adolescents, GeneXpert Ultra

## Abstract

**Objectives:**

To evaluate the contribution of Ultra to the diagnosis of pediatric pulmonary tuberculosis (PTB).

**Methods:**

We analyzed prospective data from children and adolescents with presumed PTB whose specimens were tested with Ultra between January 2020 and December 2022. Diagnosis was based on clinical-radiological criteria, clinical response after a two-month treatment period, and microbiological analysis. Ultra was considered positive with a result of ‘Detected’ and ‘Traces’ in children under 10 years and in HIV-infected individuals. Fisher’s exact test was used for group comparisons, and McNemar’s test was used to compare Ultra results with the diagnostic presumption. The study was approved by the Ethics Committee (CAAE No. 02173518.2.0000.5264).

**Results:**

A total of 41 patients were included, of whom 63% (26/41) were diagnosed with PTB. Among these, 9/26 (34%) had positive results only through Ultra, with negative AFB and culture. The sensitivity and specificity of Ultra were 50% (13/26) and 100% (15/15), respectively. The PPV was 100% (13/13), and the NPV was 54% (15/28). Of these 28 (68%) patients with negative Ultra results, 13 (46%) were diagnosed with PTB, mostly by MoH-SS. Considering culture as the reference, the PPV and NPV were 67% and 100%, respectively.

**Conclusions:**

Ultra significantly contributed to the diagnosis of pediatric PTB, proving to be a promising tool for paucibacillary forms of the disease. However, it should not be used alone. Integrating laboratory tests with clinical evaluation is essential to improving diagnostic accuracy and the management of pediatric TB.

## INTRODUCTION

Tuberculosis is a preventable and treatable communicable disease. However, it remains the world’s second leading cause of death from a single infectious agent.^1^ Children and adolescents up to 15 years old account for 11% of all tuberculosis cases, with approximately 1.1 million children diagnosed annually, half of whom are under the age of 5.^2^

National tuberculosis control programs have reported fewer than 50% of pediatric tuberculosis cases, highlighting a significant gap in case detection.^2^ This disparity largely arises because many cases involve primary tuberculosis, which is either abacillary or paucibacillary, resulting in approximately 80% of diagnoses being made without bacteriological confirmation. The challenge is further compounded by young patients’ inability to spontaneously provide sputum samples for smear microscopy.^3^ In Brazil, diagnosis has relied on the scoring system (SS) recommended by the Brazilian Ministry of Health (MoH) since 2002.^1^ However, whenever feasible, children with symptoms of pulmonary tuberculosis (PTB) should undergo initial rapid molecular testing and rifampicin resistance testing.^2^

To enhance laboratory diagnostics for tuberculosis, the World Health Organization (WHO) recommended in 2017 that the GeneXpert MTB/RIF (Xpert) rapid molecular assay be replaced with GeneXpert Ultra (Ultra) (Cepheid, CA, USA). This updated assay was adopted in Brazil in October 2019.^4^ The Ultra test has a lower detection limit for *Mycobacterium tuberculosis* (*M*. *tb*) than Xpert (15.5 vs. 116 CFU). It utilizes real-time polymerase chain reaction (PCR), incorporates two additional *M*. *tb* targets, and features changes in the fusion curve to improve rifampicin resistance detection. Results categorize the bacillary load as ‘Detected’, ‘Not detected’, or ‘Traces detected’. In people living with HIV (PLHIV), children under ten, and cases of extrapulmonary tuberculosis, trace results are considered positive.^2^ ^,^ ^5^

A systematic review and meta-analysis on pediatric tuberculosis found that the Xpert assay had a sensitivity and specificity of 64.6% and 99.0%, respectively, while the Ultra assay exhibited 72.8% sensitivity and 97.5% specificity.^6^ This review included various diagnostic standards, such as culture and a composite reference standard that combined microbiological confirmation with clinical findings, standardized according to Graham et al. (2015).^7^ Nevertheless, most studies using Ultra focus on adults, with few exploring its diagnostic accuracy in public health settings and resource-limited environments.^8^ Therefore, the aim of the present study was to evaluate the effectiveness of the Ultra assay in diagnosing pediatric PTB in a reference hospital in Rio de Janeiro (RJ), Brazil.

## METHODS

This observational, cross-sectional, descriptive study analyzed prospective data from children (ages 0-9) and adolescents (ages 10-19) with presumed PTB who were tested using the Ultra assay.^9^ ^,^ ^10^ Conducted between January 2020 and December 2022, the study took place at the Martagão Gesteira Pediatric Institute (IPPMG), a leading pediatric tuberculosis center in Rio de Janeiro (RJ), Brazil.

Eligible participants were those identified by attending physicians as having presumed intrathoracic tuberculosis (here referred to as PTB). Clinical and epidemiological data were collected through interviews with parents or legal guardians and from medical records. The analyzed parameters included age, sex, nutritional status (measured as weight percentile for age based on the Centers for Disease Control and Prevention [CDC] growth charts),^11^ MoH SS (> 40 points = very likely, 30-35 points = possible, < 25 points = unlikely),^12^ acid-fast bacilli (AFB) detection in Ziehl-Neelsen smears, *M*. *tb* culture, and human immunodeficiency virus (HIV) antibody testing.^12^

Informed consent was obtained from parents or guardians, while adolescent patients provided informed assent. Samples were then collected and sent to the Mycobacteriology Laboratory at the Clementino Fraga Filho University Hospital - Professor Newton Bethlem Institute of Thoracic Diseases (HUCFF-IDT) of the Federal University of Rio de Janeiro (UFRJ). The samples were processed for the Ultra assay, AFB testing, culture in a Mycobacteria Growth Indicator Tube (MGIT), and antibiotic sensitivity testing (AST) if the MGIT culture was positive.

All specimens were categorized as respiratory (bronchoalveolar lavage, gastric lavage, sputum, induced sputum, or string test [ST])^13^ or pleural (pleural fluid or biopsy) samples.

The final PTB diagnosis was established based on clinical-radiological criteria, clinical response two months after the start of treatment, and microbiological analysis.^1^ ^,^ ^7^ ^,^ ^14^ Children and PLHIV with trace results were considered positive.^4^ The samples were grouped into three categories: confirmed tuberculosis (Group 1), which was characterized by an MoH SS score > 30, a positive clinical response after two months of treatment, and microbiological confirmation; unconfirmed tuberculosis (Group 2), characterized by an MoH SS score > 30, a positive clinical response after two months of treatment, but no microbiological confirmation; and non-tuberculosis (Group 3), with an MoH SS score < 25, clinical improvement without PTB treatment, and no microbiological confirmation. These groupings were adapted from the classification proposed by Graham et al. (2015).^7^

The sensitivity, specificity, positive predictive value (PPV), and negative predictive value (NPV) of the Ultra assay were calculated by group, considering Groups 1 and 2 as PTB and Group 3 as non-PTB.

The data were coded and entered into a database using Excel 12.0 software (Office 2007) and analyzed using SPSS software, version 20.0, for Windows. Categorical data were assessed using descriptive statistics and expressed as frequencies and proportions. Fisher’s exact test was used for group comparisons. A p-value of less than 0.05 was considered statistically significant. McNemar’s statistical test was used to compare results from the Ultra assay with presumptive diagnoses.

This study received approval from the Research Ethics Committee (REC) of the UFRJ IPPMG, under Certificate of Submission for Ethical Appraisal (CAAE) No. 02173518.2.0000.5264.

## RESULTS

The study sample consisted of 41 patients with presumed PTB, with no exclusions. Of these, 26 (63.4%) were diagnosed with PTB (Groups 1 and 2), while 15 (36.6%) received other diagnoses (Group 3). The characteristics of the study population are presented in Table 1.

**Table 1:** Characteristics of the study population (n=41) according to final diagnosis.

	Total		Final diagnosis			
			TB (Groups 1 and 2)		Non-TB (Group 3)	
	(n=41; 100%)		(n=26; 63%)		(n=15; 37%)	
	n	%	n	%	n	%
Sex						
Male	21	100.0	11	52.0	10	48.0
Female	20	100.0	15	75.0	5	25.0
Age						
Child	21	100.0	10	48.0	11	52.0
Adolescent	20	100.0	16	80.0	4	20.0
HIV						
Positive	3	100.0	2	67.0	1	33.0
Negative*	38	100.0	24	63.0	14	37.0
Weight-for-age						
< P3	12	100.0	8	67.0	4	33.0
> P3	29	100.0	18	62.0	11	38.0
TST						
Positive	13	100.0	11	85.0	2	15.0
Negative	16	100.0	10	63.0	6	38.0
WI	12	100.0	5	42.0	7	58.0

*Including the 4 cases with no HIV record. Legend: TB, Tuberculosis; HIV, Human Immunodeficiency Virus; P3, 3rd percentile for weight-for-age; WI, Without information; TST, Tuberculin skin test.

**Figure 1:**
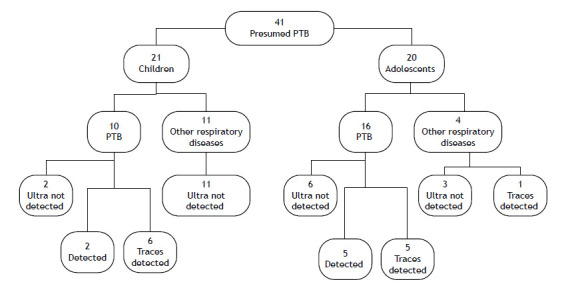
Flowchart illustrating the Ultra test results according to final diagnosis in 41 patients with presumed pulmonary tuberculosis. Legend: PTB, pulmonary tuberculosis.

The distribution of patients based on the Ultra test results is shown in Figure 1.

In Groups 1 and 2, children under 10 years and PLHIV with trace results had an Ultra assay sensitivity and specificity of 50% (13/26) and 100% (15/15), respectively. The Ultra assay demonstrated a PPV of 100% (13/13) and a NPV of 54.0 (15/28), based on a population prevalence of 64.0%. These data are shown in Table 2.

**Table 2:** Children and adolescents with presumed pulmonary tuberculosis (n=41) according to the final Ultra test results and final diagnosis.

Final Ultra test result	Total		Final diagnosis				Predictive values		p-value
			TB (Groups 1 e 2)		Non-TB (Group 3)		(for prevalence = 64%)		
	n	%	n	%	n	%	Positive	Negative	
Positive*	13	32.0	13	50.0	0	0.0	100.0%	54.0%	< 0.001
Negative	28	68.0	13	50.0	15	100.0			
Total	41	100.0	26	63.0	15	37.0			

Legend: Ultra, Molecular rapid test; TB, Tuberculosis; PTB, Pulmonary tuberculosis; Positive*, Ultra detected and traces in children under 10 years and/or PLHIV.

Quantitatively, 7/7 (100%) patients with a ‘Detected’ result and 11/12 (92%) with ‘Trace’ results were diagnosed with PTB using the Ultra assay. An adolescent with significant hematologic cancer showed trace results but was not diagnosed with PTB. This patient experienced improved respiratory symptoms within the first two weeks of treatment with common antibiotics and was, therefore, considered a false positive.

Among the 41 participants, 28 (68%) received negative results in the Ultra assay. Of these, 13 (46%) were diagnosed with PTB-12 (92%) in Group 2 and 1 (8%) in Group 1. Patients in Group 2 were diagnosed using the MoH SS. Of the 13 patients with PTB and negative Ultra assay results, 9 (82%) were categorized as very likely or possible tuberculosis, 2 (18%) as unlikely, and 2 (15%) lacked complete data for scoring.

Considering the culture as the reference standard, the PPV and NPV were 67.0% and 100%, respectively. The Ultra assay was positive in 31.0% of cases, with a specificity of 77.0% and a sensitivity of 80%, as shown in Table 3.

**Table 3:** PPV and NPV of children and adolescents with presumed tuberculosis based on final diagnosis, using culture as the reference.

Ultra	Total		Culture			
			Positive		Negative	
	n=39*		(n=5)		(n=34)	
	n	%	n	%	n	%
Detected	6	100.0	4	68.0	2	33.0
Not detected	21	100.0	0	0.0	21	100.0
Traces detected	12	100.0	1	8.0	11	92.0

*Excluding the 2 cases with contaminated cultures. Legend: Ultra, Molecular rapid test for TB; TB, Tuberculosis; PTB, Pulmonary tuberculosis.

**Figure 2:**
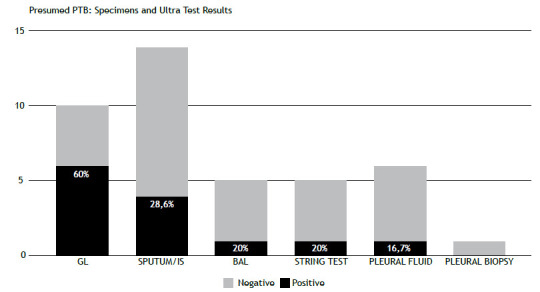
Positivity of the molecular rapid test for tuberculosis by respiratory specimen in 41 patients with PTB. Legend: IS, induced sputum; GL, gastric lavage; BAL, bronchoalveolar lavage; PTB, pulmonary tuberculosis. The percentages indicate the positivity of the Ultra test within each specimen.

In the 41 cases, 34 (83%) were respiratory and 7 (17%) were pleural, as shown in Figure 2. The Ultra assay yielded positive results in 35.0% of the respiratory samples and 14.0% of the pleural samples.

The accuracy of the Ultra assay was further assessed by comparing its results with those of concurrent positive diagnostic tests. Among the 26 (63%) patients diagnosed with PTB (Groups 1 and 2), 9 (34%) tested positive only with the Ultra assay, despite negative results from AFB and culture tests.

## DISCUSSION

This study evaluated the effectiveness of the Ultra assay in diagnosing PTB using respiratory samples from children and adolescents at a reference center in Rio de Janeiro (RJ), Brazil. Overall, 64.0% of the patients were diagnosed with PTB. The Ultra assay proved pivotal in diagnosing over one-third of the participants, even when both sputum smear microscopy and culture tests were negative. Notably, PTB was identified in half of the cases with negative Ultra assay results, highlighting that a negative mycobacterial test result does not rule out the diagnosis, thus underscoring the necessity of the MoH SS.

The absence of a definitive gold standard test remains a challenge, particularly in pediatric populations, where obtaining high-quality samples is inherently difficult. While a culture-based reference standard is commonly adopted in adults, it is less effective for diagnosing pediatric tuberculosis, as culture techniques may fail to detect up to 40% of PTB cases in this age group.^15^ Given the limitations of culture as a diagnostic tool and the variability in bacteriological confirmation, Graham et al. (2015) proposed classifying tuberculosis in children as ‘confirmed tuberculosis’, ‘probable tuberculosis’, and ‘unlikely tuberculosis.’^7^ In this study, the Ultra assay demonstrated a sensitivity of 50% and a specificity of 100% relative to the final PTB diagnosis, aligning with the findings of a 2022 Cochrane review that assessed the Ultra assay in 25,937 children under 15 years of age, reporting a sensitivity range of 23.5-50.3% and a specificity above 98.2%.^6^

Using culture as the reference standard yielded a specificity of 76.5% and a sensitivity of 80% in our population. These results are comparable to those of a prospective cohort study conducted between July 2018 and February 2019 at a tertiary hospital in northern India, which included 156 children under 15 years of age and reported a sensitivity of 85.0% (95%CI: 68.1-94.9) and a specificity of 94.0% using culture as the reference standard.^16^

The comparison of three diagnostic methods in this study-Ultra, AFB, and culture-revealed that the Ultra assay contributed to the diagnosis in just over one-third of cases where both sputum smear microscopy and culture tests were negative. This represents a significant improvement over a similar study conducted in 2019 in Rio de Janeiro, where the Xpert assay contributed to 9% of the diagnoses under similar conditions.^17^ In the present study, the diagnostic contribution of the Ultra assay increased by 25%.

Our analysis indicated that 50% of the PTB patients had negative Ultra results. Therefore, while the Ultra assay is a valuable diagnostic tool, particularly for the pediatric population, who typically present with paucibacillary tuberculosis, it should not be used alone.^2^ Our findings underscore the necessity of using the MoH SS to diagnose PTB, which has a sensitivity and specificity of 89.0% and 87.0%, respectively.^12^

A comparison of results between adolescents and children showed that PTB was confirmed in 80% (16/20) of the adolescents and 48% (10/21) of the children. The Ultra assay was positive in 31% (5/16) of adolescents and 80% (8/10) of children, with a statistically significant difference (p = 0.04). The higher positivity rate in children was attributed to trace results, which are considered positive in children under 10 years according to WHO guidelines, leading to a higher number of positive diagnoses in this age group.^6^ Conversely, adolescents had a higher rate of detectable results correlated with a positive culture. This discrepancy may be explained by the manifestation of more adult-type tuberculosis in adolescents, potentially linked to differences in bacillary load or their ability to produce higher-quality sputum samples compared to younger children.^18^

In patients with trace results, 11 out of 12 were diagnosed with PTB, with only one false positive, as previously mentioned. This finding aligns with research from South Africa, which investigated predictors of active PTB in 290 patients with trace results in the Ultra assay, including 89 children under 5 years of age. In addition to clinical interpretation, the Ultra ‘traces’ category contributed to the diagnosis of pulmonary tuberculosis.^5^

The analysis of different sample types and their Ultra assay positivity rates indicated that gastric aspirate had the highest positivity rate, at 60%, followed by sputum and induced sputum, at 28.6%. The higher positivity rate with gastric lavage, as noted in the referenced Cochrane review, may be attributed to these samples often being collected in hospital settings, where the likelihood of more advanced disease is greater.^6^

Our study had some limitations. Being a single-center study with a small sample size may have led to less precise estimates of diagnostic parameters. Additionally, as it was conducted at a tuberculosis referral center and did not include basic health units, there may be selection bias, potentially resulting in higher positive rates with the method.

Despite these limitations, this is the first Brazilian study to evaluate the Ultra assay for diagnosing PTB in an exclusively pediatric population. We concluded that the Ultra assay significantly aids in diagnosing PTB among children, proving to be a valuable tool for identifying paucibacillary forms of the disease suitable for initial screening. Although it is an important method, it should not be used alone, as a negative result does not rule out the disease.^2^ ^,^ ^12^ Laboratory tests must be complemented by clinical evaluations, and the MoH SS should be used to diagnose PTB, thereby enhancing diagnostic accuracy and improving the management of pediatric tuberculosis.
